# Massive thoracic recurrence of adrenocortical carcinoma

**DOI:** 10.1210/jcemcr/luag343

**Published:** 2026-11-06

**Authors:** Riccardo Renda, Livia Lamartina

**Affiliations:** Département D’Imagerie Médicale, Service de Cancérologie Endocrinienne, Gustave Roussy, Villejuif F-94805, France; Department of Clinical Sciences and Community Health, University of Milan, Milan 20122, Italy; Département D’Imagerie Médicale, Service de Cancérologie Endocrinienne, Gustave Roussy, Villejuif F-94805, France

**Keywords:** adrenocortical carcinoma, adrenal cancer, pulmonary metastasis

## Image legend

A man with localized left adrenocortical carcinoma underwent resection in 2008 (Weiss score 6, Ki-67 25%) and received adjuvant mitotane, which was discontinued for toxicity after 6 months [1]. A left pulmonary micronodule noted in 2018 progressed by 2024 to a 36-mm mass with hilar lymphadenopathy. Follow-up was irregular, and proposed treatment was declined. He presented to our center in 2026 at age 54 with muscle wasting, bilateral lower-limb edema, and hypertension. Hormonal work-up revealed severe adrenocorticotropic hormone (ACTH)-independent hypercortisolism: urinary free cortisol 11 × upper limit of normal (ULN) and ACTH < 3.8 pg/mL (SI: <0.84 pmol/L) (reference range, 4.7-48.8 pg/mL [SI: 1.0-10.7 pmol/L]), co-secretion of estradiol 1.7 × ULN, androstenedione 10 × ULN, 17-hydroxyprogesterone 7 × ULN, and dehydroepiandrosterone sulfate >3 × ULN. He was eupneic with normal oxygen saturation; breath sounds were absent, with left hemithorax dullness. Computed tomography revealed a massive pleuropulmonary metastasis replacing the entire left lung (Panel A), causing mediastinal shift, left pulmonary artery encasement (Panel B), pericardial invasion, and contact with the left ventricle (Panel C), plus right lung nodules (Panel D) and lymphadenopathy. Best supportive care was initiated, and the patient died 10 days later following acute respiratory deterioration. This case depicts the natural history of an untreated, initially indolent adrenocortical carcinoma progressing to extensive metastatic disease [2].

**Figure:**
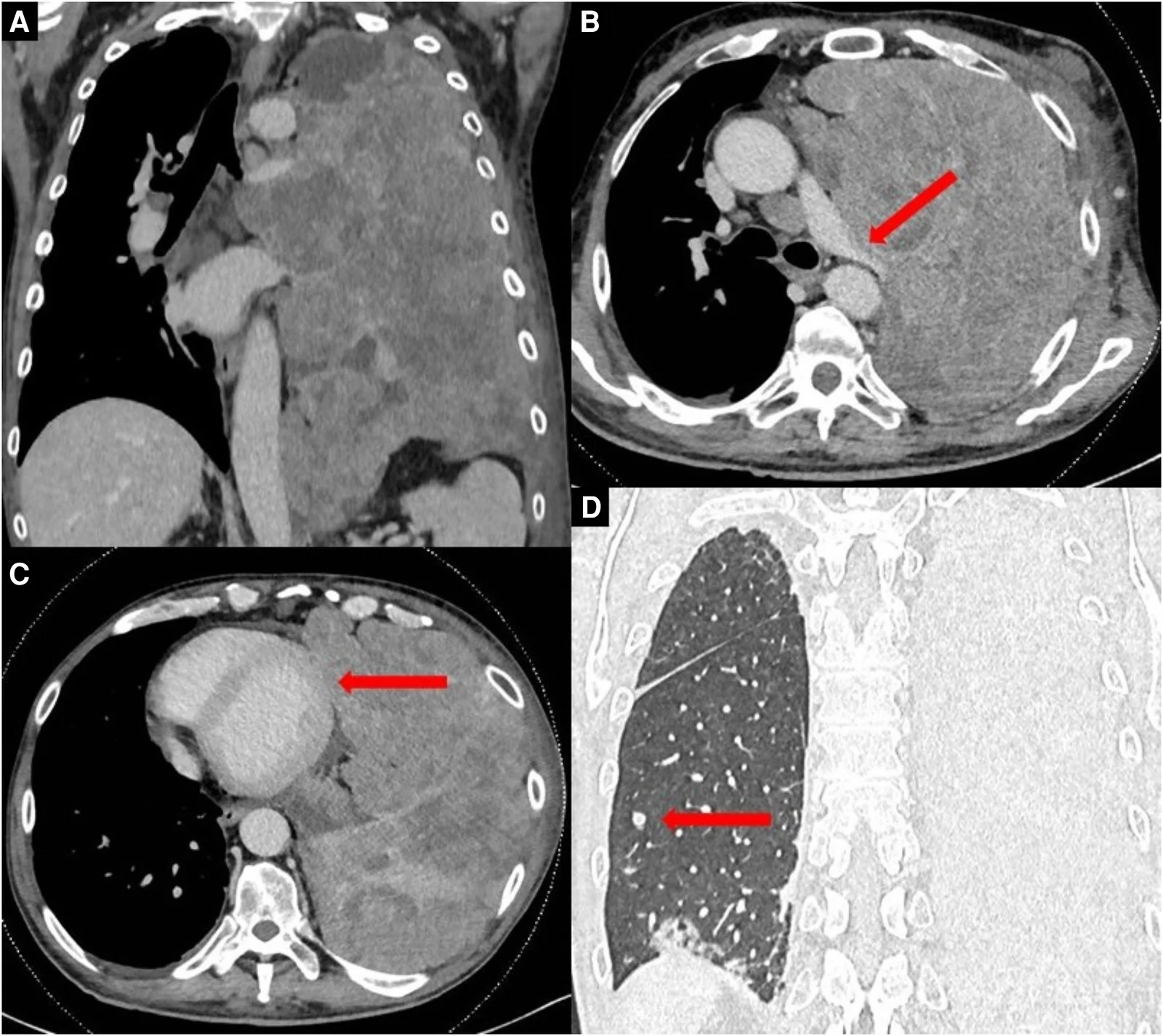


## Contributors

All authors made individual contributions to authorship. R.R. and L.L. were involved in the management of the patient and in the concept, preparation, and editing of the manuscript. Both authors reviewed and approved the final manuscript.
